# Endothelial Shear Stress and Platelet FcγRIIa Expression in Intracranial Atherosclerotic Disease

**DOI:** 10.3389/fneur.2021.646309

**Published:** 2021-02-25

**Authors:** David S. Liebeskind, Jason D. Hinman, Naoki Kaneko, Hiroaki Kitajima, Tristan Honda, Adam H. De Havenon, Edward Feldmann, Raul G. Nogueira, Shyam Prabhakaran, Jose G. Romano, Peter W. Callas, David J. Schneider

**Affiliations:** ^1^Department of Neurology, Neurovascular Imaging Research Core and UCLA Stroke Center, University of California, Los Angeles, Los Angeles, CA, United States; ^2^Department of Neurology, University of Utah, Salt Lake City, UT, United States; ^3^Department of Neurology, The University of Massachusetts Medical School-Baystate, Springfield, MA, United States; ^4^Department of Neurology, Marcus Stroke & Neuroscience Center, Emory University School of Medicine, Atlanta, GA, United States; ^5^Department of Neurology, The University of Chicago, Chicago, IL, United States; ^6^Department of Neurology, University of Miami, Miami, FL, United States; ^7^Department of Biostatistics, University of Vermont, Burlington, VT, United States; ^8^Department of Medicine, Cardiovascular Research Institute, University of Vermont, Burlington, VT, United States

**Keywords:** intracranial atherosclerosis, stroke, shear stress, FcγRIIa receptor, platelet activation and reactivity

## Abstract

Intracranial atherosclerotic disease (ICAD) has been characterized by the degree of arterial stenosis and downstream hypoperfusion, yet microscopic derangements of endothelial shear stress at the luminal wall may be key determinants of plaque growth, vascular remodeling and thrombosis that culminate in recurrent stroke. Platelet interactions have similarly been a principal focus of treatment, however, the mechanistic basis of anti-platelet strategies is largely extrapolated rather than directly investigated in ICAD. Platelet FcγRIIa expression has been identified as a potent risk factor in cardiovascular disease, as elevated expression markedly increases the risk of recurrent events. Differential activation of the platelet FcγRIIa receptor may also explain the variable response of individual patients to anti-platelet medications. We review existing data on endothelial shear stress and potential interactions with the platelet FcγRIIa receptor that may alter the evolving impact of ICAD, based on local pathophysiology at the site of arterial stenosis. Current methods for quantification of endothelial shear stress and platelet activation are described, including tools that may be readily adapted to the clinical realm for further understanding of ICAD.

## Introduction

Intracranial atherosclerotic disease (ICAD) is the most common cause of stroke worldwide (1, 2). The devastating consequences of ICAD reflect racial, sex and ethnic disparities, impact a broad age group and lack strategies for prevention (3). Overwhelming recurrent risk amounts to an excessive burden of disease and public health priority (4). ICAD engenders a ~12.5% rate of recurrent clinical strokes within 1 year (5, 6). The impact of “silent” strokes, evident only on surveillance imaging, may be even greater when one considers cognitive or other impairment.

Recurrent ischemic stroke due to ICAD is extremely common despite treatment with anti-platelet medications. Heterogeneity of the arterial architecture and associated blood flow changes in ICAD-related stenoses result in different patterns of wall shear stress (WSS) from one individual to the next. Such wall shear stress can be readily quantified with computational fluid dynamics (CFD) from non-invasive CT angiography (CTA), routinely acquired in patients with minor stroke or transient ischemic attack (TIA) due to ICAD. These shear stress changes in blood flow promote platelet aggregation and thereby alter the response to anti-platelet therapy. Additionally, greater platelet FcγRIIa expression increases platelet reactivity and promotes thrombosis when platelets are exposed to increased shear stress. In coronary artery disease (CAD), greater platelet expression of FcγRIIa identifies patients at greater risk of recurrent cardiovascular events, including stroke. Numerous mechanisms have been invoked in the recurrence of ischemia in ICAD, yet focused research on the pathophysiology of shear stress and platelet activation has not been evaluated to explain the high rate of imaging evidence and clinical strokes following minor stroke or TIA due to ICAD. Given the shared pathology of coronary artery disease and ICAD, the data suggest that individual differences in CFD-derived WSS and platelet FcγRIIa expression may inform a precision medicine strategy to prevent recurrent stroke.

## Shear-Induced Platelet Aggregation in ICAD

**Figure 1:**
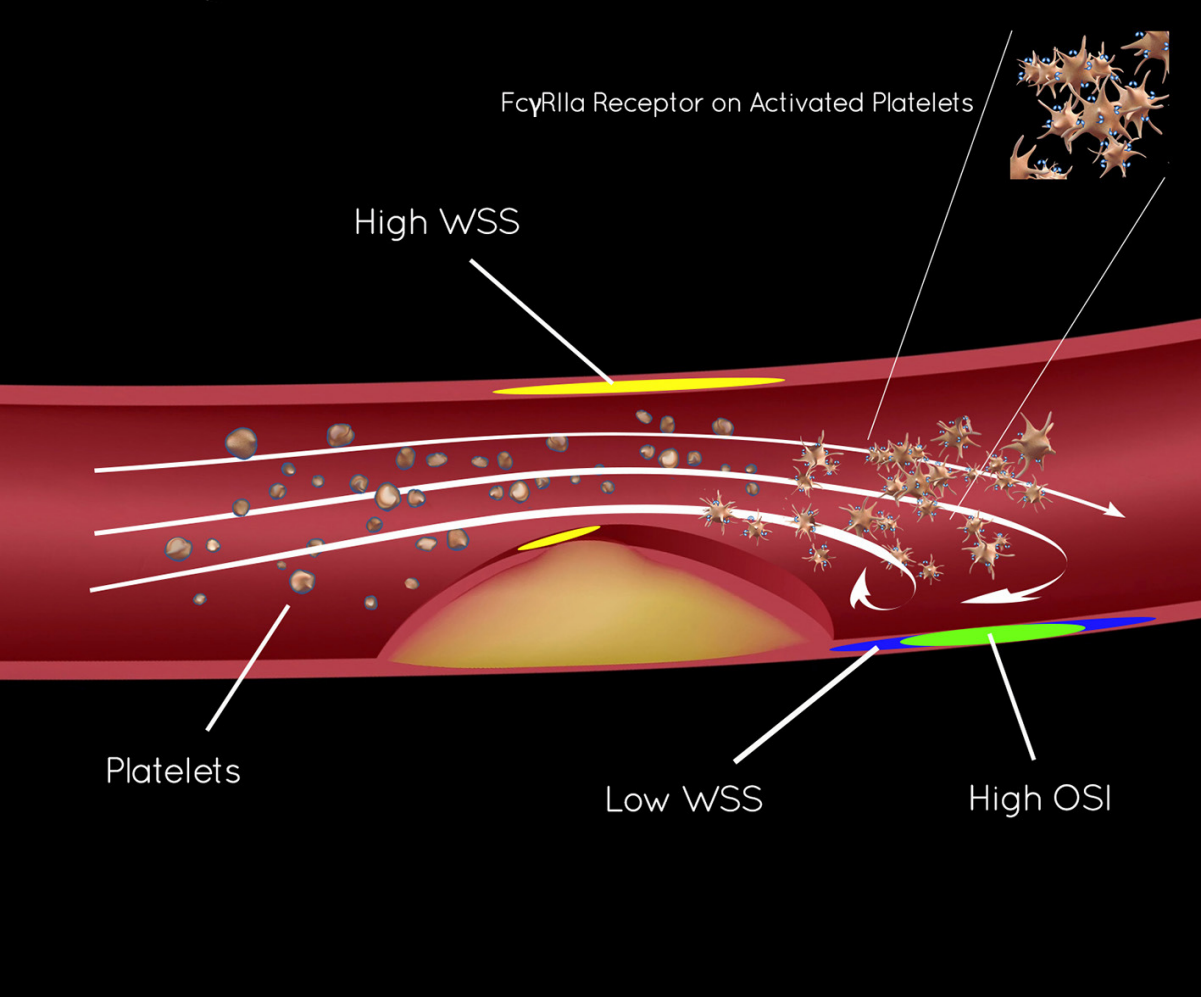
Endothelial shear stress in ICAD and activation of platelet FcγRIIa.

More than 25 years ago, stroke research underscored the pathophysiology of shear-induced platelet aggregation (7–9). *In vitro* studies showed a protective effect of thienopyridines (e.g., clopidogrel), creating parallel approaches to ICAD and CAD, based on anti-platelet effects. These studies revealed that aspirin has limited effect on platelet aggregation, modified largely by local hemodynamics, forming the rationale for dual anti-platelet therapy (DAPT) in ICAD and CAD. Distinct zones in the region of arterial narrowing or stenosis and immediately downstream in the post-stenotic segment influence platelet activation, modulated by shear stress. As in Figure 1, wall shear stress (WSS, calculated as t_s_) increases as blood flows tangentially to the arterial wall of the narrowed lumen or stenosis, measured by the residual radius. As blood flow volume asymmetrically exits the stenosis, flow vortices create oscillating gradients in both direction and intensity of WSS. High shear stress and the oscillatory shear index (OSI) can be measured with CTA techniques and are closely linked to platelet activity (10–13).

## Platelet Reactivity and Platelet Expression of FCγRIIA

Increased platelet reactivity has identified patients with minor stroke or TIA who are at greater risk of recurrent stroke (14). Similarly, increased platelet reactivity has consistently identified patients with CAD who are at greater risk of subsequent cardiovascular events (15–17). Two large clinical trials in CAD failed to demonstrate that currently available non-specific platelet function tests can be used to guide treatment (18, 19). Intra-individual variability in platelet function over time is substantial and likely to be a major contributor to the failure (20–22). Because of the failure of platelet function tests to effectively guide treatment in patients with CAD, it is unlikely that existing platelet function tests will be able to guide individualized care in ICAD.

FcγRIIa is a member of the Fc family of proteins that is expressed on the surface of platelets and amplifies platelet activation (23, 24). The Schneider Lab has pioneered the platelet biology of FcγRIIa and established it as a potential marker of risk for secondary thrombotic events in circulatory disorders. FcyRIIa amplifies activation of platelets in response to any stimulus or agonist. Importantly, platelet FcγRIIa expression amplifies thrombosis in the setting of shear forces (25). In a single center study, we found that high platelet FcγRIIa expression (≥11,000/platelet) is associated with a greater risk (odds ratio > 4) of myocardial infarction (MI), stroke and death (26). Platelet FcγRIIa expression does not require activation of platelets and does not exhibit the magnitude of intra-individual variability seen with platelet function tests (27). The emphasis on platelet activation directly focuses our stroke prevention efforts in ICAD where anti-platelets have been paramount and shear-induced platelet aggregation pivotal. FcγRIIa may identify those at high or low risk of recurrent stroke and serve as an effective tool to guide precision medicine in ICAD.

## FCγRIIA as a Marker of Platelet Reactivity and Risk of Cardiovascular Events

FcγRIIa was identified as a low-affinity receptor for the fragment constant (Fc) portion of immunoglobulin (Ig) G (28, 29). FcγRIIa markedly enhances thrombus formation when platelets are perfused over a collagen-coated flow chamber under conditions of arterial and venous shear (30). Phosphorylation of FcγRIIa amplifies the activation of platelets (23, 24). We demonstrated that platelets with more FcγRIIa exhibited greater activation in response to sub-maximal concentrations of multiple agonists (31). FcγRIIa may therefore be a novel biomarker capable of identifying patients with increased platelet reactivity. A prospective trial was designed to determine the prognostic implications of platelet FcγRIIa expression (26). Patients (*n* = 197) were enrolled shortly before discharge from hospitalization for myocardial infarction (MI, both ST elevation and non-ST elevation were included). All patients were treated with aspirin (81 mg) and treatment with clopidogrel (~64%) and ticagrelor (~36%) was balanced in patients with high and low platelet expression of FcγRIIa (26). Clinical characteristics were well-balanced with the exception of older age, diabetes, and prior revascularization being more prominent in the high expression group. Patients with platelet expression of FcγRIIa ≥11,000 had a greater risk of heart attack, stroke, and death that became apparent after 6 months. Cox regression analysis was performed and platelet expression of FcγRIIa was the sole covariate (hazard ratio 3.9, *p* = 0.035) associated with freedom from MI, stroke, and death. The sensitivity of high expression to identify patients with cardiovascular events was 0.82 (95% confidence intervals 0.57 to 0.92) and the specificity was 0.51 (95% confidence intervals 0.43 to 0.58). Cardiovascular events (heart attack, stroke, and death) were uncommon (8% of all patients experienced an event). The negative predictive value of low platelet expression of FcγRIIa was 0.97 (95% confidence intervals 0.89 to 0.98). Based on preliminary retrospective studies it has been hypothesized that a threshold of 11,000 molecules of FcγRIIa/platelet may identify high and low risk of subsequent cardiovascular events. Analysis of patients with heart attack confirmed that this threshold discriminated high and low risk most efficiently (26). As platelet expression of FcγRIIa is a continuous variable, a larger study will be required to address whether the relationship between cardiovascular events and FcγRIIa expression is continuous.

## Defining Platelet Activity and Anti-Platelet Strategies in Secondary Stroke Prevention

Anti-platelet therapies have been the mainstay of secondary stroke prevention for decades. In ICAD, “best medical therapy” is currently defined as DAPT with aspirin and clopidogrel for 90 days after stroke or TIA as in the Stenting and Aggressive Medical Management for Preventing Recurrent Stroke in Intracranial Stenosis (SAMMPRIS) trial (6). Determining platelet activity, defining long-term “best medical therapy” and establishing criteria for “failure” of anti-platelet strategies remain unaddressed. Extensive variation exists in combinations of anti-platelet strategies used and platelet activity monitoring remains a quandary. The measures in Table 1 are used sporadically, imparting bias without systematically assaying platelet activity, offering a role for FcγRIIa.

**Table 1:** Platelet assays and potential use in anti-platelet stroke prevention strategies.

**Biomarker**	**Description**	**Role**	**Pro**	**Con**
Platelet Count	• Indication of total mass of platelet	• Platelets are key to hemostasis over a wide range (150,000–400,000/μl) • Hemostasis maintained with platelet count even below 50,000/μl	• High platelet mass predisposes to exaggerated thrombosis in response to vascular injury	• Most stroke patients have normal platelet count • Increased platelet count often transient, not reflective of long-term risk
Platelet Indices	• Mean platelet volume (MPV) is a measure of platelet size • Young platelets are larger and more reactive	• Young platelets are first responders to vessel injury and critical in hemostasis • High MPV reflects more young platelets	• High MPV predisposes to exaggerated thrombosis in response to vascular injury	• High MPV in stroke patient likely due to release of new platelets after thrombosis • Increased MPV is transient, not reflective of long-term risk
Genotyping (CYP2C19)	• Genotyping for CYP2C19 will identify patients who poorly metabolize clopidogrel to form the active metabolite	• Decreased metabolism of clopidogrel to form the active metabolite leads to less antiplatelet effects	• If clopidogrel is poorly metabolized, less antiplatelet effect will occur predisposing to more events • Useful to guide alternative treatment to clopidogrel	• CYP2C19 genotyping is specific to clopidogrel • Genotyping has not been shown to predict underlying thrombotic risk
Platelet Function Testing (Verify Now)	• Measures activation of platelets in response to an agonist or combinations	• High platelet reactivity (more activation in response to an agonist) identifies subjects who are likely to have an exaggerated thrombotic response to vascular injury	• High platelet reactivity has been consistently associated with a greater risk of heart and stroke	• Platelet function tests have failed to effectively guide therapy • Platelet function tests exhibit high intra-individual variability • Platelet function tests determine response to a selected agonist/combination
Platelet FcγRIIa	• Platelet surface marker quantified with the use of flow cytometry	• Amplifies activation of platelets exposed to vessel injury/agonist/activating signal • Marker of high platelet reactivity	• Leverages implications of high platelet reactivity identified with platelet function tests • Marker of consistent increased platelet reactivity	• Requires additional validation in larger cohorts

## Arterial Hemodynamics of ICAD With CTA Computational Fluid Dynamics (CFD)

For more than a decade, routinely acquired, non-invasive CTA has been used to generate CFD measures of arterial hemodynamics in the coronary and cerebral circulations. CTA CFD has measured fractional flow reserve (FFR), elevated wall shear stress associated with arterial stenoses and post-stenotic flow aberrations, including focal areas of atherogenic low shear stress. In ICAD, almost all cases are treated with medical therapy with very few undergoing endovascular revascularization or alteration of the arterial lesion. As a result, CTA CFD can be used to characterize the local arterial hemodynamics that may predict future events. Our group has pioneered the use of CFD to quantify specific arterial hemodynamic parameters in ICAD for more than a decade (32–40). Our collaborative efforts with investigators in Beijing and Hong Kong have yielded insight on WSS in ICAD stenoses and subsequent clinical events. In a multicenter study of 245 patients (median age = 61 years, 63.7% men) we demonstrated the pivotal prognostic implication of high WSS in the stenosis (35). Stroke in the territory (SIT) occurred in 20 (8.2%) patients, mostly with multiple infarcts in the borderzone and/or cortical regions. In multivariate Cox regression, high WSS ratio (WSSR) of stenotic WSS to pre-stenotic WSS was independently associated with SIT (adjusted HR = 3.05, *p* = 0.014). These data suggest that high WSS will predict recurrent stroke, yet many other instrumental variables were not captured in that study, including post-stenotic shear force. In our most recent shear stress and endothelial pathophysiology study, we are investigating post-stenotic foci of low shear stress as a nidus for specific endothelial genotype expression, laden with atherogenic and pro-thrombotic stimuli (41). Our collaborative work integrating CFD of ICAD with microfluidic and endothelial expertise has analyzed the CTA subset acquired in the SAMMPRIS trial, showing low shear stress in the post-stenotic segment due to flow vortices proving our ability to extract and define WSS ratios at various arterial lesion sites. These retrospective analyses of SAMMPRIS are limited in ability to prove recurrent stroke due to post-stenotic low shear stress, particularly as they lack systematic MRI follow up to discern interval ischemic injury. These studies focus on only 70–99% stenoses of the proximal MCA and other potentially critical variables regarding platelet biology, anti-platelet treatment, and platelet resistance or response were not collected. *In vitro* work on shear-induced platelet aggregation strongly suggests that not just elevated WSS, but immediate downstream fluctuations in shear stress are instrumental. We have used the OSI in the post-stenotic segment to calculate, map and quantify this influential variable on *in silico* models of ICAD with CTA CFD.

## Discussion

Poor understanding of ICAD pathophysiology has been a critical barrier to progress in the field of stroke prevention. Targeting specific mechanisms of recurrent ischemia may enable clinicians to match diagnostic findings of ICAD in a given patient with the most effective therapies. Such strategies have been limited due to gaps in clinical trial design, dearth of observational studies, simplistic definitions of ICAD lesion type, empiric use of “best medical therapy,” choice of endpoints and failure to maximally leverage patient-level information from diagnostic imaging. ICAD trials increasingly focus on the most severe (70–99%) stenosis, yet almost half of ischemic strokes due to ICAD occur in milder (50–69%) lesions (42). We have previously shown that hemodynamics in ICAD are pivotal for risk stratification. Dual anti-platelet treatment (DAPT) is often used for variable durations after stroke without recognizing individual anti-platelet response or effects.

It may be possible to tackle these weaknesses using precise individual platelet biology, arterial hemodynamics of shear force across a spectrum of ICAD lesions to ascertain effect on both clinical and imaging ischemic endpoints in a multicenter cohort study. Preliminary data have established that increased platelet FcγRIIa expression is a stable measure of increased platelet reactivity. FcγRIIa can now be quantified for comparison across centers. Unlike platelet function tests, platelet FcγRIIa expression is not substantially affected by assay conditions. Finally, FcγRIIa ≥ 11,000/platelet identifies patients with ~4-fold greater risk of MI, stroke and death. Using the baseline CTA routinely acquired in our recently completed MyRIAD study, we calculated the time averaged WSS in the ICAD lesion, WSS ratio and post-stenotic OSI under pulsatile flow conditions. This preliminary research enabled us to develop standard methodology for quantification of these variables in circumferential bands of the stenosis and equivalent length post-stenosis. A larger study may extend these findings to ICAD patients, show key interplay between platelet FcγRIIa expression and WSS, providing these markers as a basis to guide individualized ICAD stroke prevention.

We have been ardently detailing a vision for precision medicine approaches to stroke and ICAD for many years now (43–56). We have described the potential of imaging features, novel assays and individual clinical characteristics of patients with acute stroke and chronic ICAD to identify therapeutic opportunities based on an *n* of 1. At a population level, we have advocated for innovative statistical methods such as clustering to discern key predictors of not just risk, but also of propensity for benefit with specific therapeutics. In retrospective analyses, we have leveraged clustering and principal component analyses to reclassify and stratify patient subsets at heightened risk of recurrent events in the datasets of past stroke randomized, controlled trials (44, 49, 57). We developed a novel approach to validate CTA CFD values of WSS in stenoses in ICAD with precision 3D cerebrovascular models, including data from the landmark SAMMPRIS trial. In other collaborations, we have separately studied the potential impact of elevated WSS on stroke recurrence in ICAD and conducted an observational multicenter study on mechanisms of recurrent stroke in ICAD. It has been demonstrated that greater platelet FcγRIIa expression increases the activation of platelets in response to agonists and shear stress. These synergies now enable investigation of how the interaction of anti-platelet therapies with individual platelet expression of FcγRIIa and WSS calculated from patient-specific CTA CFD may explain recurrent ischemia after minor stroke or TIA due to ICAD. The culmination of parallel work on shear stress-induced platelet activation in ICAD leverages preliminary data on FcγRIIa, CTA CFD of WSS and precision medicine analytics in stroke.

## Author Contributions

DL conceived and designed the manuscript, analyzed and interpreted the data, handled funding and supervision, drafted the manuscript, and made critical revision of the manuscript for important intellectual content. JH, NK, HK, TH, AD, EF, RN, SP, PC, and DS analyzed and interpreted the data and made critical revision of the manuscript for important intellectual content. All authors contributed to the article and approved the submitted version.

## Conflict of Interest

The authors declare that the research was conducted in the absence of any commercial or financial relationships that could be construed as a potential conflict of interest.
